# Letter to the Editor Re: Yamamoto R, Sugiura T, Ashida R, Ohgi K, Yamada M, Otsuka S, and Uesaka K. Prognostic value of carbohydrate antigen 19‐9 and the surgical margin in extrahepatic cholangiocarcinoma. *Ann Gastroenterol Surg*. 2022; 6:307–315

**DOI:** 10.1002/ags3.12639

**Published:** 2023-01-12

**Authors:** Gintaras Zaleskis, Dainius Characiejus, Audrius Šileikis, Paulius Bosas

**Affiliations:** ^1^ National Cancer Institute of Lithuania Vilnius Lithuania; ^2^ Center for Innovative Medicine Vilnius Lithuania; ^3^ Vilnius University, Santaros Klinikos Vilnius Lithuania

Dear Editor,

We have read with great interest a manuscript that demonstrated margin status having a significant impact on OS in the group of patients with the lowest preoperative Ca19‐9 value.^1^ In contrast, in two other groups with increased preoperative Ca19‐9, the OS rate of the R0 and R1 resection did not differ.

Here we would like to comment on the hidden message of this interesting paper referring to the data which is shown exceptionally in tables and figures but is not remarked on in the article. In the presence of minimal margins, R1 dramatically worsened the survival chances in the best prognosis group and did not have an impact on medium (B) or high‐risk (C) groups. Paradoxically, R1‐exhibiting patients in the low‐risk A group were exhibiting not only inferior OS as compared to R0 patients of their own normal group (fig. 3A) but also as compared to R1 patients in normalization (fig. 3B) or non‐normalization (fig. 3C) groups.

It seems that the authors initially aimed for an ambitious and difficult objective: to explore tumor marker Ca19‐9 reduction achieved during the surgical intervention to evaluate the completeness of resection and to predict the prognosis of EHCC. An analysis of Table 1 reveals interesting findings. The degree of Ca19‐9 reduction as a result of tumor debulking surgery was more pronounced in two higher‐risk groups (also designated as “B” and “C” in some tables) as compared to the low‐risk (A) group. The postoperative decline of the Ca19‐9 value in the normal group can be calculated as 1.6 times reduction (from 16 to 10 U/mL); whereas it exhibited a significantly better reduction—4.1 times in the highest risk group (C). Finally, the highest reduction of Ca19‐9 was seen in the B group—it exhibited 8 times decline. Nevertheless, patients in the B and C groups exhibited inferior overall survival regardless of margin status. Obviously occult distant metastasis at a time of surgical intervention in groups B and C resulted in inferior survival outcomes. The distant recurrence parameters later in time are remarkably worse in groups B and C (table 2).

We would like to offer the rate of change of Ca19‐9 in the early postoperative period to evaluate the completeness of surgical intervention. A specific growth rate (SGR) with negative values at the early postoperative period requires only two time points. Interestingly SGR can be compared between patients with no exact time points of blood sampling. The rate of tumor marker decline during an early postoperative period can signal the presence of positive margins or occult metastasis.^2^, ^4^ Similarly, SGR calculation following surgical resection for patients in remission^3^, ^4^ can be helpful in finding patients at risk of recurrence early. The monitoring of completeness of resection in oncology has been a point of debate over the past several decades.^5^ The absolute value of Ca19‐9 should be less specific and less sensitive in identifying recurrence as compared to SGR, at least in theory. We are grateful for the authors providing us with many takeaways and ideas.

## CONFLICT OF INTEREST

The authors claim no conflict of interest.

## ETHICAL STATEMENT

All the comments of this letter are in accordance with the Helsinki Declaration of 1964 and later versions.
